# Cesin, a short natural variant of nisin, displays potent antimicrobial activity against major pathogens despite lacking two C-terminal macrocycles

**DOI:** 10.1128/spectrum.05319-22

**Published:** 2023-09-27

**Authors:** Longcheng Guo, Joseph Wambui, Chenhui Wang, Francis Muchaamba, Maria Victoria Fernandez-Cantos, Jaap Broos, Taurai Tasara, Oscar P. Kuipers, Roger Stephan

**Affiliations:** 1 Department of Molecular Genetics, Groningen Biomolecular Sciences and Biotechnology Institute, University of Groningen, Groningen, the Netherlands; 2 Institute for Food Safety and Hygiene, Vetsuisse Faculty, University of Zurich, Zurich, Switzerland; Peking University Health Science Center, Beijing, China

**Keywords:** cesin, nisin, *Clostridium estertheticum*, bacteriocin, lipid II

## Abstract

Nisin is a widely used lantibiotic owing to its potent antimicrobial activity and its food-grade status. Its mode of action includes cell wall synthesis inhibition and pore formation, which are attributed to the lipid II binding and pore-forming domains, respectively. We discovered cesin, a short natural variant of nisin, produced by the psychrophilic anaerobe *Clostridium estertheticum*. Unlike other natural nisin variants, cesin lacks the two terminal macrocycles constituting the pore-forming domain. The current study aimed at heterologous expression and characterization of the antimicrobial activity and physicochemical properties of cesin. Following the successful heterologous expression of cesin in *Lactococcus lactis*, the lantibiotic demonstrated a broad and potent antimicrobial profile comparable to that of nisin. Determination of its mode of action using lipid II and lipoteichoic acid binding assays linked the potent antimicrobial activity to lipid II binding and electrostatic interactions with teichoic acids. Fluorescence microscopy showed that cesin lacks pore-forming ability in its natural form. Stability tests have shown the lantibiotic is highly stable at different pH values and temperature conditions, but that it can be degraded by trypsin. However, a bioengineered analog, cesin R15G, overcame the trypsin degradation, while keeping full antimicrobial activity. This study shows that cesin is a novel (small) nisin variant that efficiently kills target bacteria by inhibiting cell wall synthesis without pore formation.

**IMPORTANCE:**

The current increase in antibiotic-resistant pathogens necessitates the discovery and application of novel antimicrobials. In this regard, we recently discovered cesin, which is a short natural variant of nisin produced by the psychrophilic *Clostridium estertheticum*. However, its suitability as an antimicrobial compound was in doubt due to its structural resemblance to nisin(1–22), a bioengineered short variant of nisin with low antimicrobial activity. Here, we show by heterologous expression, purification, and characterization that the potency of cesin is not only much higher than that of nisin(1–22), but that it is even comparable to the full-length nisin, despite lacking two C-terminal rings that are essential for nisin’s activity. We show that cesin is a suitable scaffold for bioengineering to improve its applicability, such as resistance to trypsin. This study demonstrates the suitability of cesin for future application in food and/or for health as a potent and stable antimicrobial compound.

## INTRODUCTION

Currently, the world is faced by an increasing burden arising from antibiotic-resistant pathogens. There is an urgent need for the discovery of novel antimicrobial compounds that can be used as alternatives to or in combination with conventional antibiotics (1 – 3). Antimicrobial compounds of interest include bacteriocins, which are ribosomally synthesized peptides, that have conventionally been applied as food biopreservatives. Their potent activity against target bacteria and low toxicity toward mammalian cells make bacteriocins an attractive choice for application as either food biopreservatives or antibiotics (4, 5). A recent effort to discover novel bacteriocins from the psychrophilic *Clostridium estertheticum* complex (CEC), which has been overlooked as a source of bacteriocins, led to an unprecedented discovery of bacteriocin biosynthetic gene clusters encoding novel lantibiotics and sactipeptides (6). CEC comprises 11 closely related species that are often isolated from the meat processing environment (7 – 10). Due to their ubiquitous presence in the meat industry, CEC often contaminates and proliferates in chilled vacuum-packed meat where they cause spoilage (11 – 13). Bacteriocin production was identified as an important evolutionary trait of CEC, which was not only postulated to be critical for survival in their respective niches, but also as a resource for bacteriocins with a potential application against clinically relevant pathogens (6).

**Fig 1:**
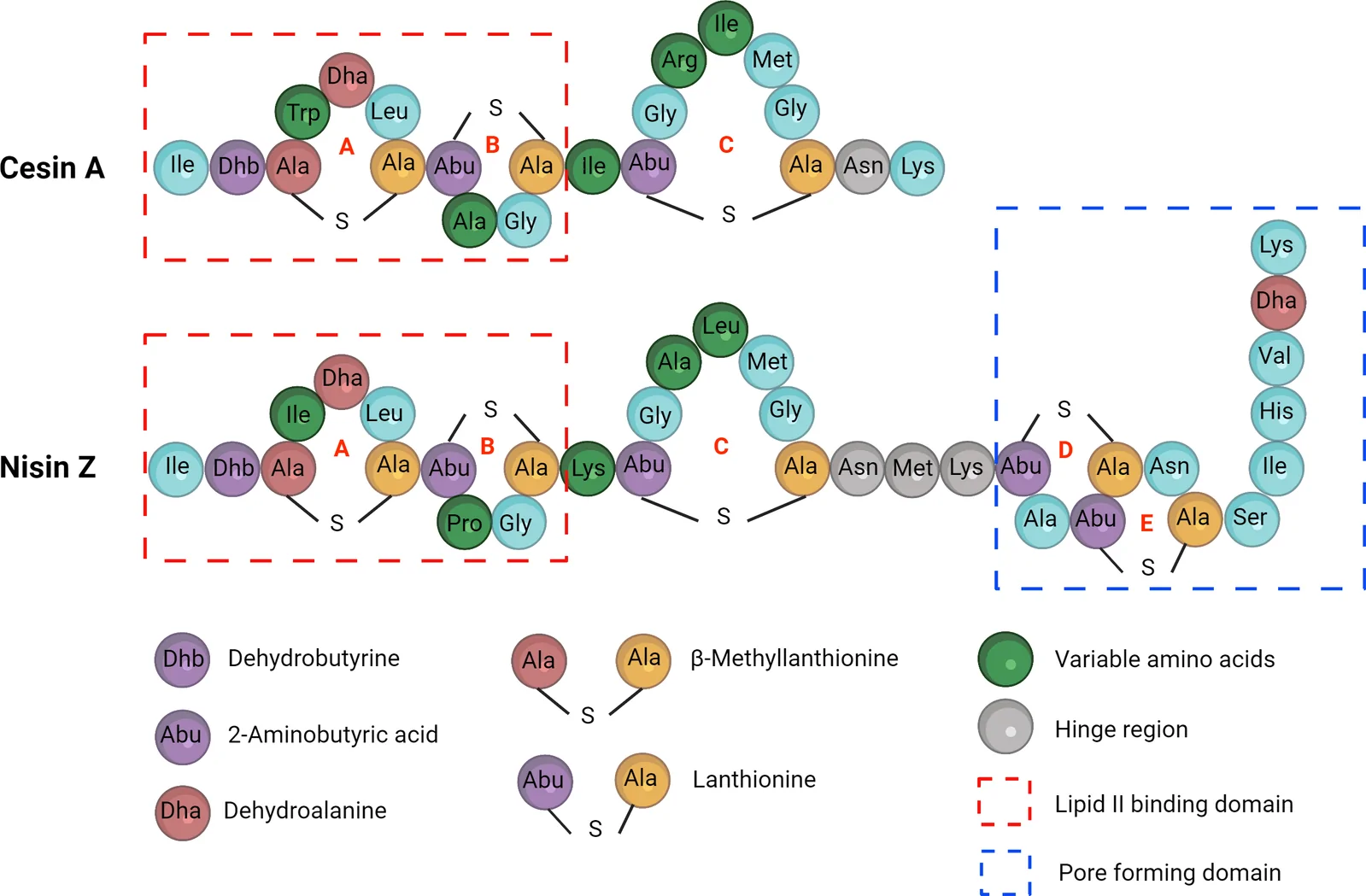
Primary structures of cesin and nisin. Regions and amino acid residues involved in the lantibiotics’ antimicrobial activity are highlighted.

Cesin A (herein referred to as cesin), which was among the six putative lantibiotics identified in CEC, is produced by multiple *C. estertheticum* strains, and is unique for its idiosyncratic structure (Fig. 1). High-resolution mass spectrometry (MS) and tandem mass spectrometry analyses revealed it is a short natural variant of nisin with 21 amino acid residues and three thioether macrocycles (6). In contrast, currently known natural variants of nisin, including nisin A, F, Q, and Z (Fig. 1) from *L. lactis* (14 – 17), nisin H, P, U, and U2 from *Streptococcus* spp. (18 – 20), nisin O from *Blautia* spp. (21), and nisin J from *Staphylococcus* spp. (22), are distinguished by their 31–35 amino acids residues and five thioether macrocycles. The two C-terminal macrocycles of nisin, referred to as rings D and E, insert into the cell membrane following the reorientation of nisin leading to pore formation in target bacteria (23 – 25). The reorientation is attributed to a hinge region between rings C and D (26 – 28). While studies have shown that the hinge region and the two macrocycles are essential for nisin’s antimicrobial activity (29), surprisingly, these regions are absent in cesin (6). Despite this, the antimicrobial activity and stability tests of partially purified extracts of a cesin-producing strain suggested the production of broad acting and stable antimicrobial compounds (6). However, these properties could not be solely attributed to cesin, since the producer strain also produced estercticin A, a bacteriocin belonging to the sactipeptide group (6). Therefore, the consequential effect of cesin’s unique structure on its antimicrobial activity remained unknown.

Bacteriocins are often extracted from the native producer strains, but in some cases, the process is cumbersome, and it is coupled with low product yields. In the case of *C. estertheticum* strains producing cesin, it takes about 4 weeks to cultivate them for bacteriocin extraction owing to their slow growth under normal laboratory conditions (6). To overcome this, heterologous expression of cesin in an alternative host such as *Lactococcus lactis* is an appealing option. The nisin-controlled expression (NICE) system in *L. lactis* is one of the commonly used systems for inducible expression of nisin and other class I bacteriocins (30). The system is advantageous given that *L. lactis* is typified by rapid growth and ease of genetic manipulation, and is generally regarded as safe (31). Recently, the NICE system was used to express lantibiotics derived from the genus *Clostridium* spp. (32), making it a suitable tool for the expression of cesin. Furthermore, the lack of genetic toolkits available for *C. estertheticum* limits the options to modify cesin through bioengineering for diversified applications. Therefore, the current study aimed at heterologous expression of cesin using the NICE system, determining its antimicrobial activity profile and mode of action in comparison to its bioengineered analogs and full-length nisin.

## RESULTS

### Cesin is effectively expressed in *Lactococcus lactis* using the nisin-controlled gene expression system

**Fig 2:**
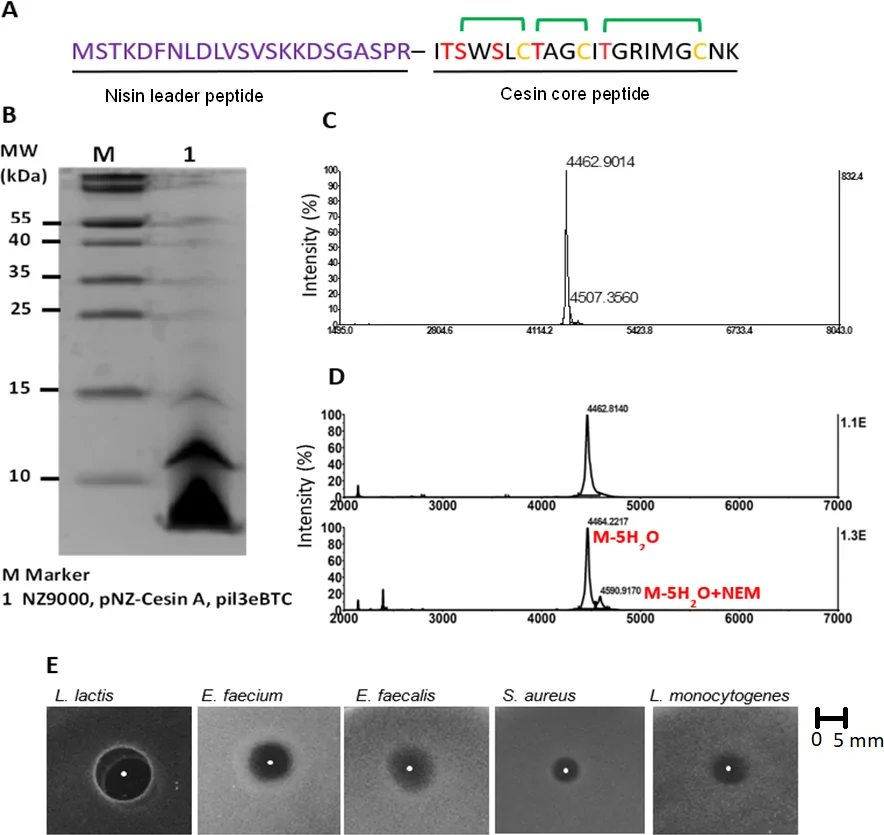
Heterologous expression of cesin in *Lactococcus lactis* using the nisin-controlled gene expression system. (**A**) The hybrid design for heterologous expression of cesin core peptide using nisin leader peptide (hybrid peptide). (**B**) SDS-polyacrylamide gel electrophoresis of the expressed hybrid peptide. (**C**) MALDI-TOF MS analysis of the hybrid peptide. The observed mass was 4,462.9 Da compared to a predicted mass of 4,459.2 Da of a modified hybrid peptide with five dehydrations. (**D**) NEM alkylation assay to determine the level of cyclization. The mass of the major peak after addition of NEM (below) was consistent with the mass of the hybrid peptide before addition of NEM (top), confirming majority of the peptide lacked free cysteines. (**E**) Antimicrobial activity assay of modified cesin core peptide after cleavage of nisin leader peptide using NisP.

The suitability of the NICE system for the heterologous expression of cesin was determined. The core peptide of cesin was fused to the nisin leader peptide (Fig. 2A) and expressed using the NICE system and the concomitant expression of *nisBTC*. An expected mass corresponding with cesin expressed with the nisin leader peptide was detected by Tricine-SDS-PAGE analysis (Fig. 2B). This was further supported by the detection of a major peak with a mass of 4,462.9 Da following matrix-assisted laser desorption/ionization time-of-flight (MALDI-TOF) analysis (Fig. 2C) that corresponds with 4,459.2 Da, which is the expected mass of a fully dehydrated cesin. This demonstrated that NisB can fully dehydrate cesin. Cesin forms three macrocycles (Fig. 1). Following the N-ethylmaleimide (NEM) alkylation assay (Fig. 2D), the major peak in the MALDI-TOF assay had a mass of 4,464.2 Da and corresponded with that of fully cyclized cesin, while the minor peak had a mass of 4,590.9 Da corresponding with an expected mass shift of 125 Da following the incorporation of NEM in one of the three free cysteines of cesin. The NEM alkylation assay showed that the bulk of the expressed cesin is fully cyclized by NisC. Antimicrobial analysis of the expressed peptide against five different strains plated on agar plates supplemented with NisP revealed inhibition zones of varying sizes (Fig. 2E), hence demonstrating the suitability of NisP to cleave the nisin leader peptide. In good agreement with the mature cesin purified from the native *C. estertheticum* producer strain, we have shown that the NICE system, together with expression of *nisBCT*, is suitable for the heterologous expression of a mature and bioactive cesin. The antimicrobial activity of the mature cesin (Fig. 2E) provided the first evidence of the broad antimicrobial activity against Gram-positive bacteria.

### With only three macrocycles, cesin displays potent antimicrobial activity

Compared to nisin Z (henceforth referred to as nisin), cesin lacks rings D and E forming the C-terminal tail (Fig. 1). The absence of these rings in a shortened nisin variant, nisin(1–22), considerably reduces the antimicrobial activity of nisin. Cesin is structurally similar to nisin(1–22) and would therefore be expected to have comparable antimicrobial activity. This prompted us to compare the antimicrobial activity of cesin with nisin and nisin(1–22) (Table 1). Surprisingly, minimal inhibitory concentration (MIC) determination against 10 Gram-positive bacteria strains from six different species showed cesin was considerably more active than nisin(1–22). Against 12 Gram-positive bacteria strains from eight different species, the activity of cesin was higher than nisin itself against *Listeria monocytogenes* LMG10470, *Staphylococcus aureus* LMG10147, and *Clostridium perfringens* CECT376 and comparable to nisin against *L. monocytogenes* strains TT82E and LK132, methicillin-resistant *S. aureus* LMG15975, *Bacillus cereus* CH-85, and vancomycin-resistant *Enterococcus faecalis* LMG16216. Nisin, on the other hand, was more active than cesin against *L. lactis* MG1363 and vancomycin-resistant *Enterococcus faecium* strains LMG16003 and LMG11423. Despite lacking rings D and E, cesin not only demonstrates broad antimicrobial activity, but its activity against important Gram-positive pathogens is, surprisingly, within the range of full-length nisin.

**TABLE 1:** Antimicrobial profile of cesin against selected Gram-positive strains in comparison to nisin and nisin(1–22)

	MIC (μg/mL)		
Bacteria	Cesin	Nisin	Nisin(1–22)
*Lactococcus lactis* MG1363	0.08	0.02	1.28
*Listeria monocytogenes* LMG10470	2.56	5.13	41.00
*Listeria monocytogenes* TT82E	5.13	5.13	10.25
*Listeria monocytogenes* LK132	10.25	10.25	41.00
*Bacillus cereus* CH-85	20.50	20.50	>41.00
*Staphylococcus aureus* LMG10147	5.13	10.25	20.50
*Staphylococcus aureus* LMG15975 (MRSA^*b*^)	5.13	5.13	20.50
*Enterococcus faecium* LMG11423	5.13	1.28	41.00
*Enterococcus faecium* LMG16003 (VRE^*c*^)	10.25	5.13	>41.00
*Enterococcus faecalis* LMG16216 (VRE)	5.13	5.13	>41.00
*Clostridium perfringens* CECT376	0.50	1.00	ND^*a*^
*Clostridioides difficile* CECT531	>31.5	>31.5	ND

^*a*^ ND, not determined.

^*b*^ MRSA, methicillin-resistant *Staphylococcus aureus.*

^*c*^ VRE, vancomycin-resistant *Enterococcus.*

### Cesin binds to lipid II and lipoteichoic acid (LTA), but lacks membrane pore-forming ability

**Fig 3:**
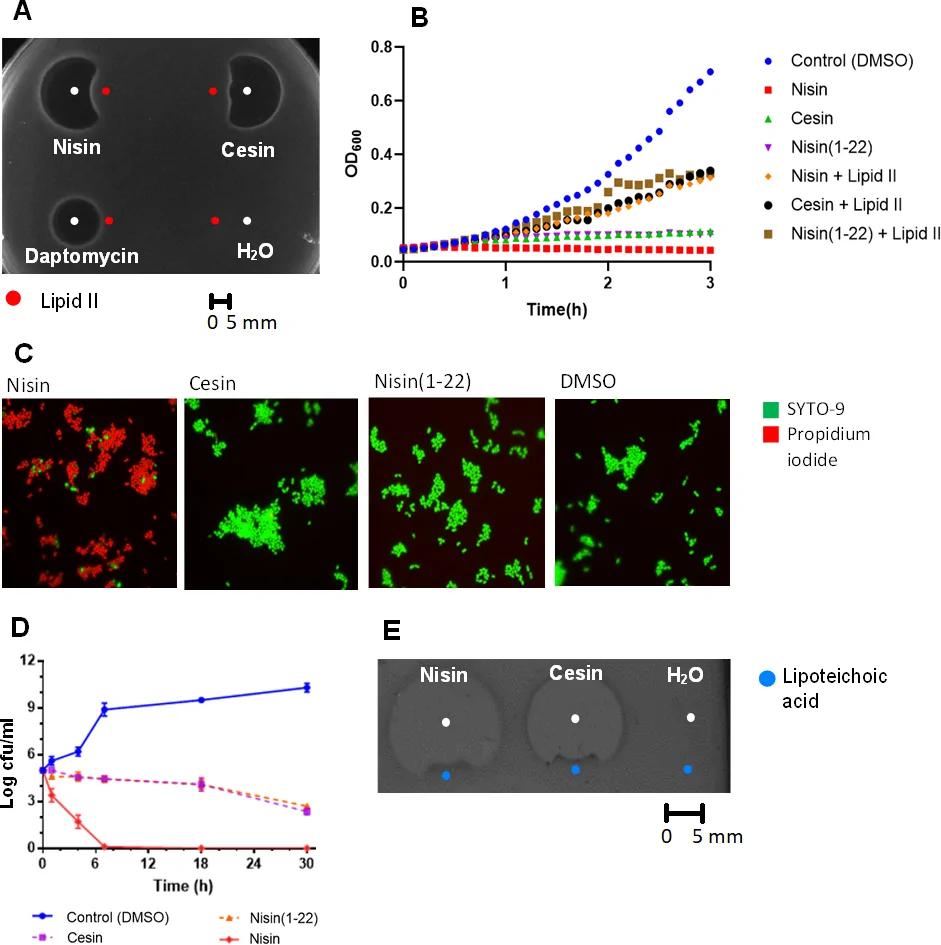
Antimicrobial mode of action of cesin in comparison to nisin against *Lactococcus lactis* MG1363. (**A**) Spot-on-lawn-based lipid II binding assay. Cesin (8 µg) and nisin (2 µg) were spotted adjacent to lipid II (300 µm, 3 µL) and the antimicrobial activity determined. Daptomycin (2 µg) and water were used as controls. (**B**) Growth curve-based lipid II binding assay. Cesin, nisin, and nisin(1–22) (5 × MIC) and 2 µL lipid II (0.6 mol/L) were added and microbial growth was monitored through spectrophotometry. DMSO was used as a control. (**C**) Membrane pore-forming ability of cesin, nisin, and nisin(1–22) (2 × MIC) in microbial cells were determined using a combination of microscopy and fluorescent dyes, SYTO-9 (membrane permeable), and propidium iodide (membrane impermeable). (**D**) Time-killing curves of cesin, nisin, and nisin(1–22) (10 × MIC) against the bacteria. (**E**) Spot-on-lawn-based LTA binding assay. Cesin and nisin (8 µg) were spotted adjacent to LTA (1 mg/mL, 3 µL) and the antimicrobial activity. Water was used as a control.

The primary mode of action of many lanthipeptides is inhibition of peptidoglycan synthesis by binding to lipid II. The lipid II binding domain is conserved between cesin and nisin (Fig. 1); therefore, to determine the mode of action of cesin, we initially determined its lipid II binding ability (Fig. 3A). Consistent with its structure, cesin bound to lipid II, as demonstrated with reduced antimicrobial activity against *L. lactis* MG1363. Similar observations were made for nisin, but not daptomycin whose antimicrobial activity does not involve lipid II binding. Further evidence of lipid II binding was provided through growth curves (Fig. 3B). The addition of lipid II to cesin reduced its activity and a similar effect was observed for nisin and for nisin(1–22). Nisin also inhibits target bacteria by inducing membrane pore formation, a process that is attributed to the hinge region and rings D and E and a tail ending with a positive Lys residue (Fig. 1). Apart from the terminal Lys residue, these domains are absent in cesin and nisin(1–22). Therefore, we used a combination of fluorescence microscopy using two different dyes, the green fluorescent SYTO-9 and the red fluorescent propidium iodide (PI), to determine the pore-forming ability of cesin (Fig. 3C). The SYTO-9 binds to DNA and emits fluorescence by penetrating both intact and damaged membranes while the PI only binds to DNA and emits fluorescence after penetrating damaged membranes. The red PI was observed after *L. lactis* MG1363 was exposed to nisin, which is consistent with its membrane pore-forming ability. Conversely, only the green dye was observed when the strain was exposed to cesin or nisin(1–22) demonstrating that the cell membrane was still intact. This demonstrated that cesin lacks pore-forming ability, which can be attributed to the lack of the two terminal macrocycles essential for pore formation. The data show that cesin lacks a dual mode of action that typifies other natural variants of nisin. Pore formation by nisin usually corresponds with rapid dissipation of transmembrane electrostatic potential resulting in membrane permeabilization and rapid bacterial cell death. The killing assay (Fig. 3D) demonstrated rapid cell death when *L. lactis* MG1363 was treated with nisin. This observation was not made when either cesin or nisin(1–22) was used. The slower rate of killing observed for cesin than nisin is thus consistent with its predicted mode of action that does not involve membrane pore formation. Cesin has two positively charged amino acids, R15 and K21, but no negatively charged amino acids; hence at neutral pH, it has a net positive charge similar to other natural nisin variants. The net positive charges modulate the sensitivity of target Gram-positive bacteria through electrostatic interactions with net negatively charged teichoic acids. The LTA binding assay (Fig. 3E) showed that the antimicrobial activity of cesin against *L. lactis* MG1363 was reduced after the addition of LTA. Similar observations were made for nisin. Therefore, cesin also uses electrostatic interactions to interact with the cell wall of target bacteria.

### Alteration of cell wall surface charge through the deletion of *dltA* gene increases sensitivity of *Listeria monocytogenes* to cesin

**Fig 4:**
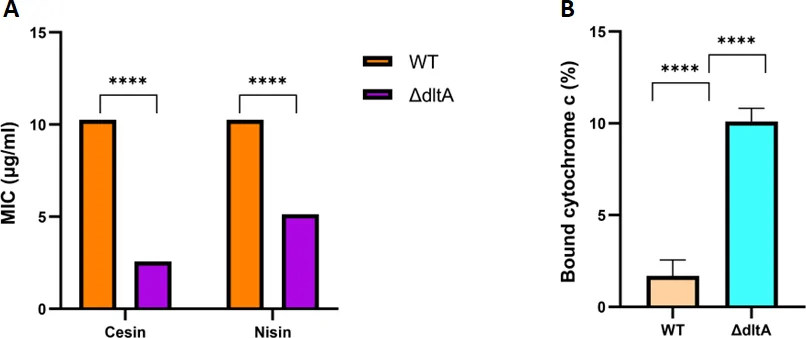
Effects of the *dltA* (*lmo0974*) gene deletion in *L. monocytogenes*. (**A**) The *ΔdltA* mutant has increased sensitivity to both cesin and nisin than the WT strain. (**B**) The *ΔdltA* mutant has increased binding affinity to the positively charged cytochrome c than the WT strain. The data support interaction between the positively charged cesin and negatively charged cell wall components.

We have shown that cesin, like nisin, uses electrostatic charges to interact with the cell wall by binding to LTA (Fig. 3E). We hypothesized that disruption of the D-alanylation process, which is important for increasing the net positive charges of the LTA and wall teichoic acids in a target Gram-positive bacteria such as *L. monocytogenes*, will sensitize the bacteria to cesin and provide further evidence of the electrostatic interactions between the bacteriocin and cell wall components. Consistently, a knockout mutant of *dltA* in *L. monocytogenes* EGDe was more sensitive to the lantibiotic as well as nisin than the wild-type (WT) strain (Fig. 4A). A Δ*dltA* mutant has a higher electronegative charge than the WT which allows it to bind to positively charged compounds, such as cytochrome c and nisin, with high affinity (33). As anticipated, the cytochrome c binding assay (Fig. 4B), which is an estimation of binding affinity of the cell envelope to the positively charged lantibiotics, showed the Δ*dltA* bound more cytochrome c than the WT. Combined, these data support further validating the electrostatic interactions between cesin and cell wall components.

### Addition of two terminal macrocycles from nisin reduces the potency of cesin

**Fig 5:**
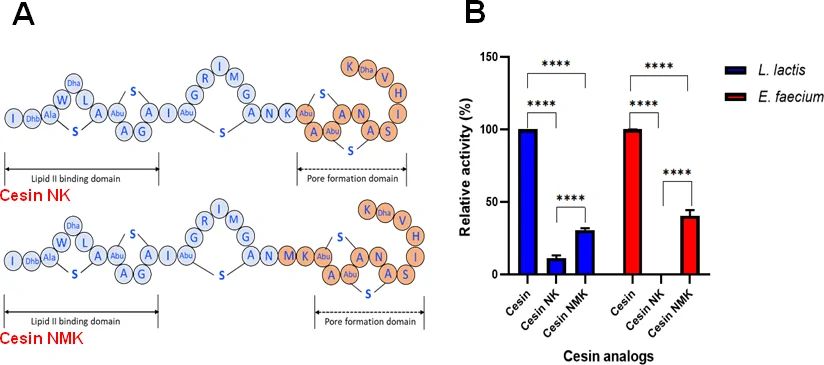
Bioengineered analogs of cesin with the C-terminal pore-forming domains of nisin. (**A**) Top: cesin NK is a hybrid of cesin and nisin(23–34). Bottom: cesin NMK is a hybrid of cesin(1–20) and nisin(21–34). (**B**) Antimicrobial activity of the bioengineered analogs of cesin against *L. lactis* MG1363 and *E. faecium*.

Having shown that cesin lacks pore-forming ability, we set out to establish the effect of complementing it with the two terminal macrocycles of nisin by bioengineering two cesin-nisin chimeras (Fig. 5A), cesin NK (cesin-nisin(23–34) hybrid) and cesin NMK (cesin(1–20)-nisin(21–34) hybrid). Both chimeras showed a significant loss of antimicrobial activity compared to that of cesin (Fig. 5B). Although the two chimeras were considerably less active, cesin NMK had significantly higher antimicrobial activity compared to cesin NK. Cesin NMK introduces the hinge region of nisin, which aids in pore formation. We therefore showed that unlike nisin, the three macrocycles of cesin, which are formed by rings A, B, and C (Fig. 1), are sufficient for its full potency. Further efforts to introduce the pore-forming domain of plantaricin C through the creation of the cesin-plantaricin C chimeras also resulted in reduced antimicrobial activity against *L. lactis* and *E. faecium* (Fig. S1), confirming the uniqueness of cesin.

### W4 and K21 are important for cesin’s antibacterial potency

**Fig 6:**
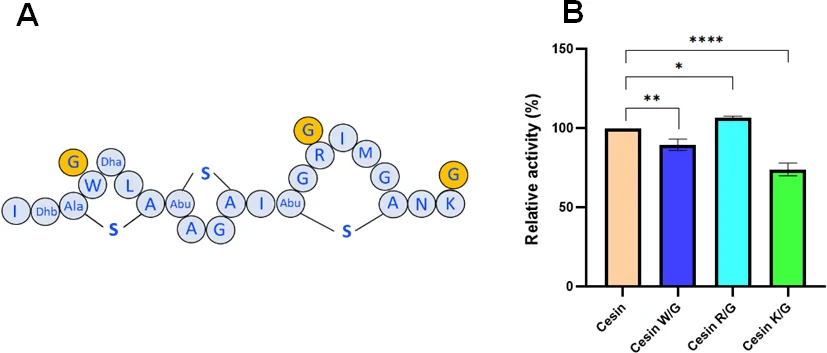
Antimicrobial activity of cesin analogs against *L. lactis* MG1363. Cesin W/G, cesin R/G, and cesin K/G analogs were bioengineered after W4G, R15G, and K21G amino acid substitution, respectively.

The cesin amino acid sequence differs in the lack of residues 22–34 and also in the first 21 amino acids of nisin by five amino acids, namely W4, A9, I12, R15, and I16, which correspond to I4, P9, K12, A15, and L16 in nisin. Previous studies have demonstrated that substitution of P9 with alanine (P9A) in nisin did not affect its antimicrobial activity (34). Substitution of K12 with isoleucine (K12I) in nisin resulted in a minor decrease in activity (11% reduction) (35). Mutants of nisin, such as L16A, L16H, and L16V, in which L16 was replaced with other hydrophobic residues, showed increased antimicrobial activity by approximately 11%–13% (36). W4 and R15 are unique to cesin among the currently characterized class I lantibiotics. We hypothesized that the unique properties of W4 and R15, including hydrophobicity and positive charge, respectively, could confer the unique properties to cesin and consequently influence its antimicrobial activity. We also hypothesized that a terminal lysine has a functional role due to its presence in most lantibiotics (Fig. 1). To determine the functional roles of these three amino acids, we created cesin W4G, R15G, and K21G analogs (Fig. 6A). Cesin W4G and cesin K21G showed significantly reduced antimicrobial activity against *L. lactis* compared to cesin (Fig. 6B). In contrast, cesin R15G showed slightly but significantly higher antimicrobial activity against the bacteria (Fig. 6B). Therefore, W4 and K21 are important for the antimicrobial activity of cesin, while R15 might have other unknown functional roles.

### Cesin displays high thermal and pH stability comparable to nisin

**Fig 7:**
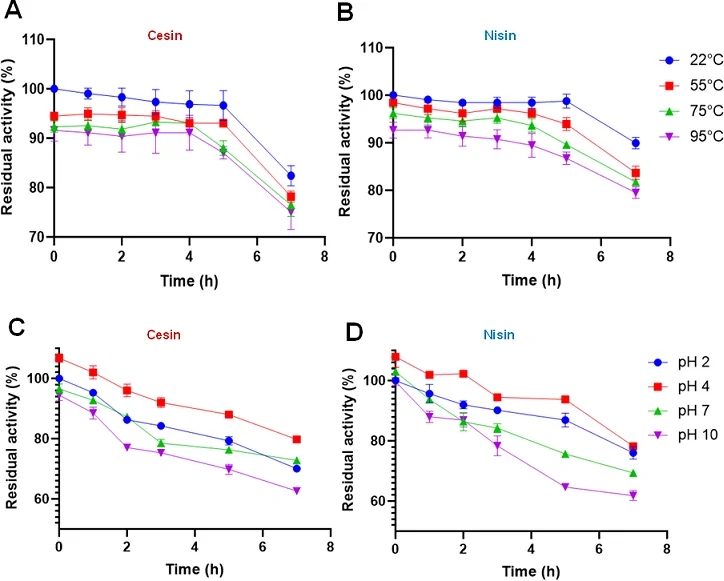
Thermal stability of cesin (**A**) compared to nisin (**B**). pH stability of cesin (**C**) compared to nisin (**D**).

The widespread application of nisin is partly due to its stability against different harsh conditions, although it is sensitive to NSR (nisin resistance protein) that cuts in the C-terminal tail. Because of this, we compared the stability of cesin to nisin when exposed to various temperatures, pHs, and proteases (Fig. 7). Both lantibiotics were stable for up to 4 h at between 22°C and 95°C, with activity being reduced by less than 30% after 7 h (Fig. 7A and B). Compared to its activity at pH 4, which was the optimum for both lantibiotics, the antimicrobial activity reduced gradually over time as the pH was reduced to 2 or increased to 7 and 10, although they retained about 60% of their activity after 7 h (Fig. 7C and D). Collectively, the data show that cesin has a thermal and pH stability comparable to that of nisin.

### Cesin is resistant to the nisin resistance protein, but it is inactivated by trypsin

**Fig 8:**
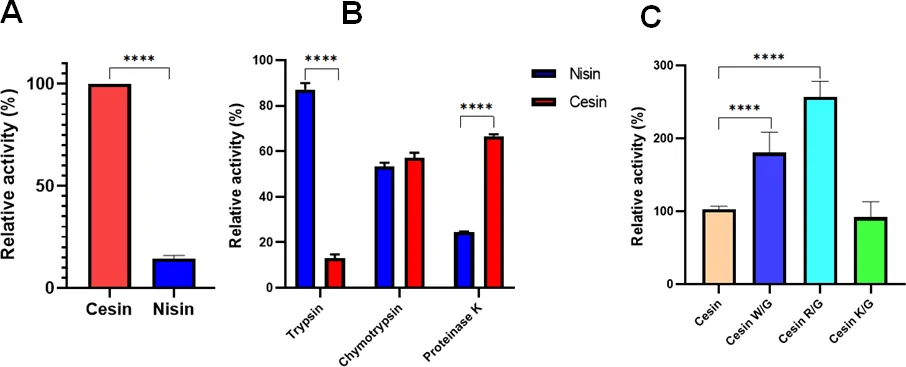
Variations in stability of cesin and nisin to proteolytic cleavage. Relative antimicrobial activity of the lantibiotics after exposure to (**A**) nisin resistance protein and (**B**) different proteolytic enzymes. (**C**) Residual antimicrobial activity of cesin and its analogs W/G, cesin R/G, and cesin K/G with glycine substitution of W4, R15, and K21, respectively, relative to cesin after exposure to trypsin.

A major limitation of application of lantibiotics is degradation by proteolytic enzymes. Specifically, nisin is cleaved by the NSR of non-nisin-producing lactic acid bacteria, thus reducing its activity. Therefore, we initially compared the resistance of cesin and nisin to NSR. Not surprisingly, we found that cesin was fully resistant to the cleavage action of NSR, while nisin was inactivated (Fig. 8A). This is consistent with the cleavage action of NSR that cleaves nisin at the peptide bond between MeLan28 and Ser29 in the C-terminus, a domain that is absent in cesin. Further analysis showed that both cesin and nisin had varying stability against trypsin and proteinase K but not chymotrypsin (Fig. 8B). Specifically, cesin was more resistant to proteinase K, but more sensitive to trypsin than nisin.

### The R15G amino acid substitution improves the stability of cesin against trypsin

Trypsin is a common enzyme in mammals and its degradation effect on cesin would dramatically reduce the potential of developing the lantibiotic further as an antimicrobial agent. We assumed that the Arg residue unique to cesin is targeted by trypsin and thus is responsible for the observations in Fig. 8B. We therefore tested the stability of the cesin R15G analog (Fig. 6A) against trypsin. The analog showed a significant increase in tolerance to trypsin (Fig. 8C), showing that the analog not only had higher antimicrobial activity than cesin and the other analogs (Fig. 6B), but it is also a suitable analog for future development as an antimicrobial agent in conditions where trypsin is present.

### Cesin has low hemolytic activity that is comparable to that of nisin

**Fig 9:**
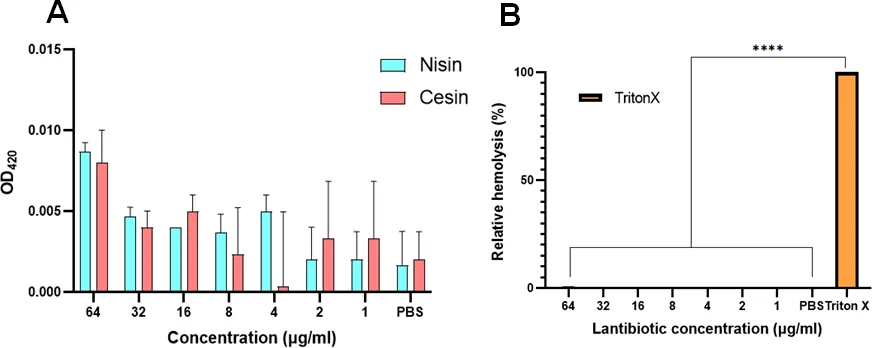
Hemolytic activity of cesin in comparison to nisin. (**A**) The release of hemoglobin from the sheep blood erythrocytes incubated with cesin and nisin at concentrations ranging from 1 to 64 µg/mL. Cells treated with phosphate-buffered saline (PBS) only were used as no-lysis controls. (**B**) The level of hemolysis of cesin and nisin relative to 10% Triton X-100, which was used as the positive control.

To assess the safety of cesin for other potential applications, its hemolytic activity against sheep erythrocytes was determined and compared to that of nisin (Fig. 9). Cesin showed a low level of hemolysis even at the relatively high concentration of 64 µg/mL. The level of hemolysis induced by cesin was comparable to that of nisin suggestive of similar safety levels (Fig. 9).

## DISCUSSION

Cesin (Fig. 1) is a recently discovered lantibiotic from the psychrophilic *C. estertheticum*, and it is currently the shortest naturally occurring variant of nisin (6). Structurally, cesin resembles nisin(1–22), a bioengineered short variant of nisin with considerably lower antimicrobial activity than full-length nisin due to the lack of the hinge region and rings D and E (29). Although cesin bears a striking structural resemblance to nisin(1–22), we assume that its unique structure has been optimally enhanced over time through evolution for its inherent biological purposes, including potent antimicrobial activity. Until now, no functional or molecular data were available for the lantibiotic. This prompted us to characterize its antimicrobial activity and potential as a therapeutic agent.

In order to further study cesin and unlock opportunities for its bioengineering and application, a suitable expression system was first needed. This is based on the notion that molecular techniques are lacking for *C. estertheticum*. Furthermore, the majority of species within the *Clostridium* spp. genus are not amenable to genetic manipulation (37). Previous studies have pioneered expression of class I lantibiotics of *Clostridium* spp. origin using the nisin-inducible expression (NICE) system and expression of *nisBTC* genes (32). Consequently, we have leveraged on the nisin modification system’s promiscuity (38) and demonstrated its suitability to express cesin (Fig. 2A) expediting the characterization and further modifications of the novel lantibiotic. Cleavage of the nisin leader peptide *in vitro* revealed cesin could inhibit a broad range of Gram-positive strains (Fig. 2E). Although cesin demonstrated broad antimicrobial activity, its potency was initially taken in doubt due to its structural resemblance to nisin(1–22). However, the true potency was confirmed following MIC determination (Table 1), whereby cesin was not only more active than nisin(1–22), but also displayed either higher or comparable antimicrobial activity to the full-length nisin against 7 out of 12 tested Gram-positive strains, including the clinically relevant, *C. perfringens*, methicillin-resistant *S. aureus* , and *E. faecalis* (VRE) (Table 1). This indicated that cesin could find applications in the gut, e.g., to fight *C. perfringens* infections, in particular if it would be less prone to proteolytic degradation (see below). These findings firmly urged the need for further characterization of cesin to fully explain the observations.

Nisin has a dual mode of action against target bacteria that involves lipid II binding. First, it inhibits cell wall synthesis by displacing lipid II from the septa (39) then causes membrane pore formation following lipid II-induced transmembrane reorientation (40 – 42). Lipid II binding occurs within the pyrophosphate cage where rings A and B of nisin physically interact with the pyrophosphate moiety (43). The reorientation of nisin in membranes from parallel to perpendicular with respect to the membrane surface and subsequent membrane pore formation is mediated by the hinge region and rings D and E (28). Structurally, rings A and B of cesin and nisin are conserved (Fig. 1). Consistent with its structure, the ability of cesin to also bind to lipid II has been demonstrated presently (Fig. 3A and B) and can be linked to the two rings. Consistent with the functional roles of nisin’s hinge region and rings D and E, we have shown that cesin lacks membrane pore-forming ability (Fig. 3C). Our efforts to bioengineer cesin with the pore-forming domains of nisin (Fig. 5) and plantaricin C (Fig. S1) decreased its antimicrobial activity. These observations led us to conclude that the unique sequence and structure of cesin are optimally designed for enhanced antimicrobial activity without the need for membrane pore formation. This is particularly supported by the decreased antimicrobial activity of cesin W4G (Fig. 6B). W4 is unique to cesin among the currently characterized class I lantibiotics, suggesting an important role in lipid II-cesin interaction similar to the role played by W1 in subtilin (44). The inability of cesin to form membrane pores can be linked to its small structure which prevents it from completely spanning the cytoplasmic membrane. This is consistent with the 22-residue class I lantibiotics, gallidermin and epidermin (45), and the 20-residue class II lantibiotic, mersacidin (46) that also lack pore-forming ability due to their small size, although their ring topologies are completely different from those of cesin. These properties distinctively differentiate cesin from other known naturally occurring members of the nisin family. We therefore propose that cesin’s mode of action, after its N-terminus binds to lipid II, follows the carpet model. In this model, antimicrobial peptides accumulate and orientate parallel to the membrane, and after a critical saturation level is reached, permeabilization occurs via global bilayer destabilization (47, 48).

Using *L. monocytogenes*, which is among the most naturally nisin-resistant Gram-positive pathogens (49), we have shown that deletion of *dltA* gene increased the susceptibility of the bacteria to cesin by fourfolds (Fig. 4A). The *dltA* gene, which is part of the *dlt* operon, codes for the D-alanine:D-alanyl carrier protein ligase, an enzyme that is involved in D-alanylation of the teichoic acids (50, 51). Inhibition of the gene increases the susceptibility of target bacteria to positively charged antimicrobial compounds that target the cell wall as a result of reduced net positive charges of the cell wall (52, 53). This is consistent with the present data where we have shown the *dltA* mutant bound more of the positively charged cytochrome c than the wild-type strain (Fig. 4B). Therefore, besides lipid II binding, electrostatic interactions are important for sensitizing target bacteria to cesin. This is also evident in the cesin K/G which had reduced antimicrobial activity. The terminal lysine, which is positively charged, is important for the lanthipeptides antimicrobial activity through electrostatic interactions with the negatively charged cell wall components.

Stability of bacteriocins against different conditions is essential for diversified application. We have found that cesin with its three macrocycles maintains a temperature and pH stability profile similar to nisin, although pH changes had a more profound effect than temperature changes on both lantibiotics (Fig. 7). The decrease in activity of cesin at higher or lower pH can be extrapolated from observations made for nisin in relation to the unsaturated Dha residues (54). At low pH, a water molecule can be added to the two unsaturated Dha residues of nisin leading to production of amide and keto acids, which can ultimately result in the peptide being cleaved at positions 5 and/or 33. At high pH, nucleophiles can be added to the unsaturated residues leading to polymerization or cleavage of the peptide (54). Although cesin lacks the Dha at position 33 due do the absence of the two terminal macrocycles, Dha at position 5 is conserved and may therefore be an important factor affecting the chemical stability of the novel nisin variant. On the other hand, the observed variable resistance to the nisin resistance protein (Fig. 8A) and proteolytic enzymes (Fig. 8B) can be attributed to differences in the amino acid sequence of both lantibiotics. With particular emphasis on trypsin, we have shown that the Arg at position 15 has a considerable effect on the proteolytic action of the protease and the new analog, cesin R15G, in which the Arg is substituted with Gly (Fig. 6A), improves both antimicrobial activity (Fig. 6B) and stability against trypsin (Fig. 8C). These observations are consistent with previous reports that trypsin cleaves exclusively C-terminal to arginine and lysine residues (55). Cesin R15G shows that cesin presents a unique scaffold that can be bioengineered for improved and diversified applications. Gly was chosen as a replacement amino acid instead of the more commonly used Ala in order to maintain good flexibility in the chain to allow for correct circularization. However, other residues can also be considered in future studies to further optimize relevant properties of cesin. The suitability of cesin for diversified applications is further enhanced by its low level of hemolysis, which is comparable to that of nisin (Fig. 9). Nisin is considered safe for use due to its low level of toxicity to mammalian cells (56). Further studies to assess the safety levels of cesin in a similar context are currently being pursued by our group.

### Conclusion

Cesin is the first natural variant of nisin that, despite lacking two commonly present macrocycles, possesses broad and potent antimicrobial activity. Although devoid of membrane pore-forming ability, the potency of cesin is attributed to lipid II binding and electrostatic interactions with the teichoic acids. Cesin also possesses similar stability as nisin to temperature and pH, making it appealing for application in food processing. Although the lantibiotic can be degraded by trypsin, we have engineered a new analog, cesin R15G, which is resistant to trypsin enlarging the application options of the lantibiotic. Further characterization of cesin including *in vivo* and *in vivo* toxicity assays as well as the detailed response of target bacteria will improve the efficacy of this novel lantibiotic for diverse applications.

## MATERIALS AND METHODS

### Heterologous expression and purification of cesin

#### Bacterial strains, plasmids, and growth conditions

Strains and plasmids used in this study are listed in Table S1. *L. lactis* NZ9000 was used for plasmid construction, plasmid maintenance, and peptide expression. All *L. lactis* strains were routinely cultured in M17 broth supplemented with 0.5% (wt/vol) glucose at 30°C. Five micrograms per milliliter of erythromycin and/or chloramphenicol was added where necessary. For protein expression, overnight cultures were inoculated (40-fold diluted) on minimal expression medium (MEM) and induced by 8 ng/mL nisin at an optical density at 600 nm (OD_600_) of about 0.4.

#### Construction of expression vectors

Oligonucleotide primers used for cloning, mutations, and sequencing in the present study are listed in Table S2. All the primers were ordered from Biolegio B.V. (Nijmegen, The Netherlands). Constructs coding for designed peptides or mutations were made by amplifying the template plasmid using a phosphorylated downstream sense (or upstream antisense) primer and an upstream antisense (or downstream sense) primer. The DNA amplification was carried out by using phusion DNA polymerase (Thermo Fisher Scientific, Waltham, MA). Self-ligation of the resulting plasmid was carried out with T4 DNA ligase (Thermo Fisher Scientific, Waltham, MA). The electrotransformation of *L. lactis* was carried out as previously described using a Bio-Rad gene pulser (Bio-Rad, Richmond, CA) (57). The designed peptide or mutations were verified by sequencing using the primer pNZ sequencing externally by Macrogen Europe B.V.

#### Precursor peptide expression and precipitation

*L. lactis* NZ9000 cells with pIL3eBTC were transformed with peptide-encoding plasmid (100 ng), plated on GM17 agar plate supplemented with chloramphenicol (Cm, 5 µg/mL) and erythromycin (Em, 5 µg/mL), and grown at 30°C for 24 h. A single colony was used to inoculate 4 mL of GM17CmEm broth. The culture was grown overnight at 30°C and then used to inoculate 20 mL (40-fold dilution) of MEM. When the OD_600_ reached 0.4–0.6, 8 ng/mL nisin was added to induce peptide expression. After induction at 30°C for 3 h, the culture supernatants were harvested by centrifugation at 8,000 rpm for 20 min. The precursor peptides were precipitated by trichloroacetic acid (TCA) for further analysis as previously described (58).

#### Screening the antibacterial activity of peptides by spot-on-lawn assay

Overnight-cultured strains were added to 0.8% Luria–Bertani (LB) agar medium or GM17 agar (w/v, temperature at 45°C) at a final concentration of 0.1% (v/v), and then 10 mL mixture was poured onto the plate. Subsequently, 5 µL precursor peptide plus 1 µL NisP spotted on the agar. After the solution drops had dried, the plates were incubated overnight at 37°C (except for *L. lactis* MG1363 at 30°C).

#### Tricine-SDS-PAGE analysis

The precipitated precursor peptides were analyzed by Tricine-SDS-PAGE as previously described (59). Briefly, 10 µL of each sample mixed with 8 µL loading dye was loaded on the gel, and then the gel was stained with Coomassie Brilliant Blue G-250.

#### Evaluation of (methyl)lanthionine formation

Cesin samples were dissolved in 16 µL of phosphate-buffered saline (pH 7.4); the samples were treated with 2 µL of 5 mg/mL tris(2-carboxyethyl)phosphine for 30 min at room temperature. Subsequently, the samples were treated with 4.5 µL of 25 mg/mL N-ethylmaleimide. After incubation at room temperature for 2 h, the samples were desalted by C_18_ ZipTip (Millipore) according to the manufacturer’s instructions and analyzed by MALDI-TOF MS.

#### Purification of cesin and nisin(1–22)

To obtain pure peptides for activity assays, the supernatant of 2 L cultures were first incubated with purified NisP (60) for 6 h at 37°C to cleave off the nisin leader, and then the supernatant was loaded onto the C_18_ open column (Spherical C_18_, particle size: 40 μm–75 μm, Sigma-Aldrich). The column was washed with 50 mL different concentrations of buffer B (buffer A, water with 0.1% trifluoroacetic acid (TFA); buffer B, acetonitrile with 0.1% TFA). The active fractions were lyophilized and stored at −20°C for further analysis. Lyophilized samples were dissolved in 65% acetonitrile with 0.1% TFA then purified by high-performance liquid chromatography (HPLC) using an Agilent 1200 series HPLC with an RP-C_12_ column (Jupiter 4 µm Proteo 90A, 250 × 4.6 mm, Phenomenex). The peaks corresponding with the fully modified peptide, correct molecular weight, and showed activity were lyophilized and stored at −20°C until further use.

#### MALDI-TOF MS characterization

MALDI-TOF MS analysis was performed using a 4800 Plus MALDI-TOF/TOF analyzer (Applied Biosystems) in the linear-positive mode. Briefly, 1 µL sample was spotted on the target plate, dried at room temperature, then washed several times with Milli-Q water on the target. Subsequently, 1 µL of 5 mg/mL of α-cyano-4-hydroxycinnamic acid was spotted on each sample, dried at room temperature, then analyzed.

### Physiochemical and antimicrobial characterization of cesin

#### Agar well diffusion assay

An overnight culture of test strains was added at 0.1% (vol/vol) into melted GM17 agar at 45°C and poured in plates. After agar solidification, wells of 8 mm were bored and filled with 30 µL of the lantibiotic solution. When necessary, the lantibiotic was activated with 5 µL of nisP added directly into the well. Peptides were resuspended in 0.05% aqueous acetic acid solution and the cesin amount was determined using the NanoDrop (Thermo Scientific) calibrated with the extinction coefficient predicted by ExPASy (http://web.expasy.org/protparam/). The agar plates were incubated at 30°C overnight, after which, the zones of inhibition were measured. Zone diameters were measured in millimeters and recorded as area of the zone (πr^2^) minus the area of the well (πr^2^) in millimeters. Where necessary, cesin was compared with nisin and nisin(1–22). Assays were performed in triplicate.

#### Effects of enzyme, pH, temperature, and nisin resistance protein on antibacterial activity

The effect of proteolytic enzymes, pH, and temperature on antimicrobial activity of cesin was carried out using representative strains *L. lactis* MG1363 and compared with nisin. Thirty microliters of peptides were directly added into the agar well to a final concentration of 1 mg/mL proteolytic enzymes (pH 7.2). The pH stability was determined by adjusting the pH to 2, 4, 7, and 10 using 1M HCl or NaOH. The temperature stability was determined by incubating the peptides at 22°C, 55°C, 75°C, and 95°C for indicated hours. To compare the resistance levels of nisin and cesin to the NSR, 30 µL 0.1 mg/mL of purified peptides were directly added to the agar well bored in agar seeded with *L. lactis* NZ9000 (pNSR, NSR-positive strain) and *L. lactis* NZ9000 (pEmpty, NSR-negative strain) as target indicators. All experiments were carried out in three biological replicates.

#### MIC and time-kill assay

MIC of cesin in comparison to nisin and nisin(1–22) was evaluated by broth microdilution according to the standard guidelines using cation-adjusted Mueller-Hinton broth (61). Briefly, the culture inocula were adjusted to approximately 5 × 10^5^ colony-forming unit (CFU)/mL and then challenged with peptide concentrations ranging from 0.02 µg/mL to 41 µg/mL. All assays with *Clostridium* were done using Reinforced Clostridium Medium (Sigma-Aldrich) and performed in a Coy Anaerobic Chamber. The MIC was defined as the lowest concentration of antimicrobial compound with no visible growth after 16- to 24-h incubation at 37°C (except for *L. lactis* MG1363 at 30°C). Time-kill assays for the three peptides were carried out according to a previously described protocol (62). Briefly, an overnight culture of *L. lactis* MG1363 was diluted 50-fold in GM17 and incubated at 30°C. Bacteria were grown to OD_600_ of 0.5, and then the cell concentration was adjusted to 5 × 10^5^ CFU/mL. Bacteria were then challenged with 10-fold MIC of each peptide. Untreated cell suspension was used as a control. At indicated time points, 50 µL aliquots were taken and undiluted, and 10-fold serially diluted suspensions were plated on GM17 agar. After incubating overnight at 30°C, colonies were counted then calculated as the colony-forming units per milliliter. Both assays were performed in triplicate.

#### Qualitative peptide-lipid II and peptide-lipoteichoic acid complex formation

An overnight culture of *L. lactis* MG1363 was added to 0.8% GM17 agar (wt/vol, temperature 45°C) at a final concentration of 0.1% (vol/vol), and then, 10 mL of this mixture was pour-plated. The binding of cesin (8 µg) and other test antimicrobials (nisin and daptomycin, 2 µg) to purified lipid II (0.6 mol/L) was tested by spot-on-the-lawn assay. Briefly, 5 µL antimicrobials were spotted on the agar plate. After drying, 2 µL lipid II was spotted to the edge of inhibition halo of antibiotics. After the lipid II solution drops had dried, the plate was incubated at 30°C overnight. The binding of cesin and nisin to teichoic acids was determined using lipoteichoic acid (1 mg/mL, 3 µL) (LTA) from *Bacillus subtilis* (Sigma-Aldrich, St. Louis, MO, USA) using the same procedure as lipid II. Three biological repeats were carried out.

#### Growth curve and lipid II binding assay

Cultures of *L. lactis* MG1363 grown overnight were diluted to an OD_600_ of 0.05 in a 96-well plate with a final volume of 200 µL per well and incubated in a microplate spectrophotometer at 30°C. When the OD_600_ reached 0.1, five-fold MIC value concentration of peptides [cesin, nisin and nisin(1–22)] was added to each well, and the same volume of DMSO was used as a control. Two microliters of lipid II (0.6 mol/L) was added to investigate the association with peptides. Growth of the bacteria was continued under the same conditions and recorded periodically for 3 h using the same microplate spectrophotometer. Three replicates were used for each treatment.

#### Fluorescence microscopy assay

*L. lactis* MG1363 was grown to an OD_600_ of 0.8. Cells were collected by centrifugation at 8,000 g for 5 min and the cell pellet was washed three times in 0.7% NaCl. After normalization of the cell density to an OD_600_ of 0.2 with 0.7% NaCl, a two-fold MIC value concentration of peptides [cesin, nisin and nisin(1–22)] was added to the cell suspension simultaneously with pre-mixed SYTO 9 and propidium iodide (LIVE/DEAD Baclight Bacterial Viability Kit, Invitrogen). After incubation at room temperature in darkness for 20 min, the tested compounds were removed by washing the cells three times in 0.7% NaCl. Subsequently, the cell suspensions were loaded on 1.5% agarose pads and analyzed by a DeltaVision Elite microscope (Applied Precision).

### Construction of *L. monocytogenes* Δ*dltA* mutant

The EGDe Δ*dltA* mutant was created through in-frame deletion of the *dltA* (*lmo0974*) gene. A deleted copy of the gene retaining the reading frame, first 6 and last 10 codons, and 500 bp of the upstream and downstream flanking sequences, was synthesized from GenScript Biotech, Netherlands, based on the *L. monocytogenes* EGDe genome sequence. The synthesized Δ*dltA* fragment was seamlessly cloned into the pKSV7 plasmid vector (63) using the In-Fusion cloning system (Takara Bio SAS Europe, Saint-Germain-en-Laye, France). The resulting plasmid was used to replace the chromosomal copy of the gene in *L. monocytogenes* EGDe strain by homologous recombination as previously described (64). The EGDe *dltA* gene loci deletion mutant was confirmed through PCR analysis and DNA sequencing. The MIC of the *ΔdltA* and WT strains was determined after exposure to both cesin and nisin as described above.

### Cytochrome c binding assay

Changes in the cell surface electrostatic charges were determined among *L. monocytogenes* EGDe WT and Δ*dltA* strains as previously described (33). Briefly, overnight cultures of the strains grown at 37°C were diluted (1:100) in brain heart infusion (BHI) broth and grown to an OD_600_ of 1.0. The cultures were harvested by centrifugation (8,000 × *g* for 5 min) and washed twice (8,000 × *g* for 5 min) with 20 mM MOPS [3-(N-morpholino)propanesulfonic acid] buffer (pH 7) (Sigma-Aldrich Co., Missouri, USA). After washing, the cells were standardized to an OD_600_ of 0.25 in MOPS buffer, and then cytochrome c (Sigma-Aldrich, St. Louis, MO, USA) was added at a concentration of 50 µg/mL. The mixture was incubated in the dark for 15 min at room temperature. After incubation, the OD_530_ of the samples was determined (OD_530_ with cells) followed by centrifugation (13,000 × *g* for 5 min). The supernatant was collected, and its OD_530_ was measured (OD_530_ without cells). Amount of cytochrome c bound by the strains was calculated and expressed as a percentage as follows: % bound cytochrome c = [(OD_530_ cells)/(OD_530_ without cells)] × 100.

### Hemolytic activity assay

The hemolytic activity of cesin and nisin was determined as previously described (65). Briefly, fresh sheep blood (Sigma-Aldrich) was washed with 0.1 M PBS (pH 7.4; Sigma-Aldrich) three times to remove plasma (500 × *g* for 5 min), and then the erythrocytes were collected and diluted 1:50 in PBS. The lantibiotics were then added at final concentrations of 64, 32, 16, 8, 4, 2, and 0 µg/mL in PBS to wells containing 2% (vol/vol) washed erythrocytes. The cells were incubated at 37°C for 40 min, then centrifuged for 5 min at 3,100 g. The supernatants were transferred to a 96-well plate, and the absorbance was measured at a wavelength of 420 nm with a Synergy HT microplate reader (BioTek, Lucerne, Switzerland). The absorbance relative to the positive control, which was treated with 0.1% Triton X-100, was defined as the percentage of hemolysis. The analysis was carried out in triplicate.

### Statistical analysis

Data analysis was carried out with GraphPad Prism 9.4.1 (GraphPad Software, CA, USA). Differences in means (*P* ≤ 0.05) were calculated using two-tailed Student’s *t*-test or two-way analysis of variance multiple comparisons whereby ns, *, **, ***, and **** corresponded with *P* > 0.05, *P* < 0.05, *P* < 0.01, *P* < 0.001, and *P* < 0.0001, respectively.

## SUPPLEMENTAL MATERIAL

Supplemental file 1 spectrum.05319-22-s0001.docx Supplemental figures and tables.

ASM does not own the copyrights to Supplemental Material that may be linked to, or accessed through, an article. The authors have granted ASM a non-exclusive, world-wide license to publish the Supplemental Material files. Please contact the corresponding author directly for reuse.
