# Supplementary material for: Cesin, a short natural variant of nisin, displays potent antimicrobial activity against major pathogens despite lacking two C-terminal macrocycles
Source: Microbiol Spectr. 2023 Sep 27;11(5):e05319-22. doi: 10.1128/spectrum.05319-22 (PMC10581189; doi:10.1128/spectrum.05319-22)
Supplement: Supplemental file 1 — Supplemental figures and tables. [file spectrum.05319-22-s0001.docx]

**Supplementary files**

**Table S1. Strains and plasmids used in this study**

| **Strains or plasmids** | **Characteristics** | **Source** |
| --- | --- | --- |
| **Strains** |  |  |
| *Lactococcus lactis* NZ9000 | Plasmid construction, plasmid maintenance, and peptide expression | Kuipers et al., 1997 |
| *L. lactis* NZ9000 (pNZ-empty) | Indicator strain | MOLGEN* Lab collection |
| *L. lactis* NZ9000 (pNZ-SV-SaNSR) | Indicator strain, coding the nisin resistance peptidase | Zaschke-Kriesche et al., 2019 |
| *L. lactis* MG1363 | Indicator strain | MOLGEN Lab collection |
| *Listeria monocytogenes* LMG10470 | Indicator strain | MOLGEN Lab collection |
| *L. monocytogenes* TT82E | Indicator strain | ILS^¥^ Lab collection |
| *L. monocytogenes* LK132 | Indicator strain | ILS Lab collection |
| *Bacillus cereus* CH-85 | Indicator strain | ILS Lab collection |
| *L. monocytogenes* EGDe | Indicator strain | ILS Lab collection |
| *L. monocytogenes* EGDe *ΔdltA* | Mutant strain | ILS Lab collection (This study) |
| *Staphylococcus aureus* LMG10147 | Indicator strain | MOLGEN Lab collection |
| *S. aureus* LMG15975 | Indicator strain, MRSA | MOLGEN Lab collection |
| *Enterococcus faecium* LMG11423 | Indicator strain | MOLGEN Lab collection |
| *E. faecium* LMG16003 | Indicator strain, VRE | MOLGEN Lab collection |
| *E. faecalis* LMG16216 | Indicator strain, VRE | MOLGEN Lab collection |
| *Clostridium perfringens* CECT376 | Indicator strain | MOLGEN Lab collection |
| *Clostridioides difficile* CECT531 | Indicator strain | MOLGEN Lab collection |
|  |  |  |
| **Plasmids** |  |  |
| pIL3EryBTC | *nisBTC*, encoding nisin modification machinery, EryR | van Heel et al., 2013 |
| pNZ–nisA (1-22) | *nisA* (1-22), encoding NisA from first amino acid to 22nd, under the control of PnisA promoter | Zhao et al., 2020 |
| pNZ–nisA | *nisA*, encoding NisA, under the control of PnisA promoter | van Heel et al., 2013 |
| pNZnisP8H | *nisP*, encoding NisP mutant, with 8 histidines, CmR | Montalbán-López et al., 2018 |
| pNZ–Cesin | Encoding cesin, under the control of PnisA promoter | This study |
| pNZ–Cesin (W/G) | Cesin mutation, the core peptide 4th amino acid tryptophan changed to glycine | This study |
| pNZ–Cesin (R/G) | Cesin mutation, the core peptide 15th amino acid arginine changed to glycine | This study |
| pNZ–Cesin (K/G) | Cesin mutation, the core peptide 21st amino acid lysine changed to glycine | This study |
| pNZ–Cesin-NK | Cesin nisin hybrid peptide, core sequence ITSWSLCTAGCITGRIMGCNKTATCHCSIHVSK | This study |
| pNZ–Cesin-NMK | Cesin nisin hybrid peptide, core sequence ITSWSLCTAGCITGRIMGCNMKTATCHCSIHVSK | This study |
| pNZ–Cesin-ktkk | Cesin plantaricin c hybrid peptide, core sequence  ITSWSLCTAGCITGRIMGCNKKTKK | This study |

*Department of Molecular Genetics, Groningen Biomolecular Sciences and Biotechnology Institute, University of Groningen, Groningen, The Netherlands

^¥^Institute for Food Safety and Hygiene, Vetsuisse Faculty, University of Zurich, Zurich, Switzerland

**References**

Kuipers, O. P., de Ruyter, P. G. G. A., Kleerebezem, M., & de Vos, W. M. (1997). Controlled overproduction of proteins by lactic acid bacteria. Trends in Biotechnology, 15(4), 135–140. https://doi.org/10.1016/S0167-7799(97)01029-9

Montalbán-López, M., Deng, J., van Heel, A. J., & Kuipers, O. P. (2018). Specificity and application of the lantibiotic protease NisP. Frontiers in Microbiology, 9(FEB), 160. https://doi.org/10.3389/FMICB.2018.00160/BIBTEX

van Heel, A. J., Mu, D., Montalbán-López, M., Hendriks, D., & Kuipers, O. P. (2013). Designing and producing modified, new-to-nature peptides with antimicrobial activity by use of a combination of various lantibiotic modification enzymes. ACS Synthetic Biology, 2(7), 397–404. https://doi.org/10.1021/SB3001084/SUPPL_FILE/SB3001084_SI_001.PDF

Zaschke-Kriesche, J., Behrmann, L. v., Reiners, J., Lagedroste, M., Gröner, Y., Kalscheuer, R., & Smits, S. H. J. (2019). Bypassing lantibiotic resistance by an effective nisin derivative. Bioorganic & Medicinal Chemistry, 27(15), 3454–3462. https://doi.org/10.1016/J.BMC.2019.06.031

Zhao, X., Yin, Z., Breukink, E., Moll, G. N., & Kuipers, O. P. (2020). An Engineered Double Lipid II Binding Motifs-Containing Lantibiotic Displays Potent and Selective Antimicrobial Activity against *Enterococcus faecium*. Antimicrobial Agents and Chemotherapy, 64(6). https://doi.org/10.1128/AAC.02050-19/SUPPL_FILE/AAC.02050-19-S0001.PDF

**Table S2. Primers for PCRs used in this study**

| **Name** | **Templates** | **primers** | **Nucleic acid sequences (5' to 3')** | **Characteristics (5'-lable)** |
| --- | --- | --- | --- | --- |
| pNZ–Cesin | pNZ–nisA | pNZ-1 | ACAGGAAGGATTATGGGTTGTAACAAATAAGCTTTCTTTGAACCAAAATTAG | 5'- phosphorylation |
|  |  | pNZ-2 | AATACAACCTGCTGTACATAGCGACCAACTTGTAATGCGTGGTGAT |  |
| pNZ–Cesin (W/G) | pNZ–Cesin | pNZ-3 | GGTTCGCTATGTACAGCAGGTTGT |  |
|  |  | pNZ-4 | ACTTGTAATGCGTGGTGATGC | 5'- phosphorylation |
| pNZ–Cesin (R/G) | pNZ–Cesin | pNZ-5 | GGTATTATGGGTTGTAACAAATAAGCTTTCTTTG |  |
|  |  | pNZ-6 | TCCTGTAATACAACCTGCTGTACA | 5'- phosphorylation |
| pNZ–Cesin (K/G) | pNZ–Cesin | pNZ-7 | GGTTAAGCTTTCTTTGAACCAAAATTAGAAAACC |  |
|  |  | pNZ-8 | GTTACAACCCATAATCCTTCCTGTAATAC | 5'- phosphorylation |
| pNZ–Cesin-NK | pNZ–Cesin | pNZ-9 | AGTATTCACGTAAGCAAA TAAGCTTTCTTTGAACCAAAATTAGAAAACC | 5'- phosphorylation |
|  |  | pNZ-10 | ACAATGACAAGTTGCTGTTTTGTTACAACCCATAATCCTTCCTGTAATA |  |
| pNZ–Cesin-NMK | pNZ–Cesin | pNZ-11 | AGTATTCACGTAAGCAAA TAAGCTTTCTTTGAACCAAAATTAGAAAACC | 5'- phosphorylation |
|  |  | pNZ-12 | ACAATGACAAGTTGCTGTTTTCATGTTACAACCCATAATCCTTCCTGTAATACAA |  |
| pNZ–Cesin-ktkk | pNZ–Cesin | pNZ-13 | AAAACAAAGAAA TAAGCTTTCTTTGAACC |  |
|  |  | pNZ-14 | TTTGTTACAACCCATAAT | 5'- phosphorylation |
| pNZ-sequencing |  |  | TATGAGATAATGCCGACTGTACTTTTTAC |  |


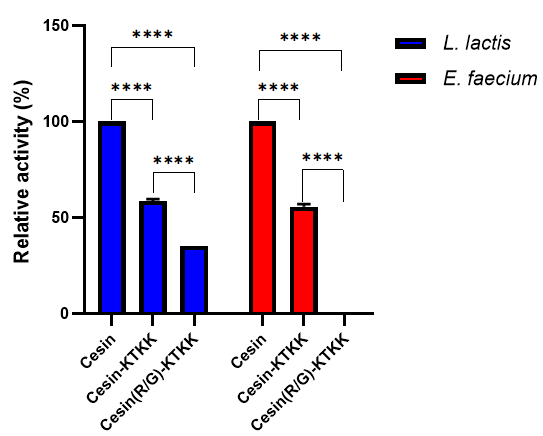

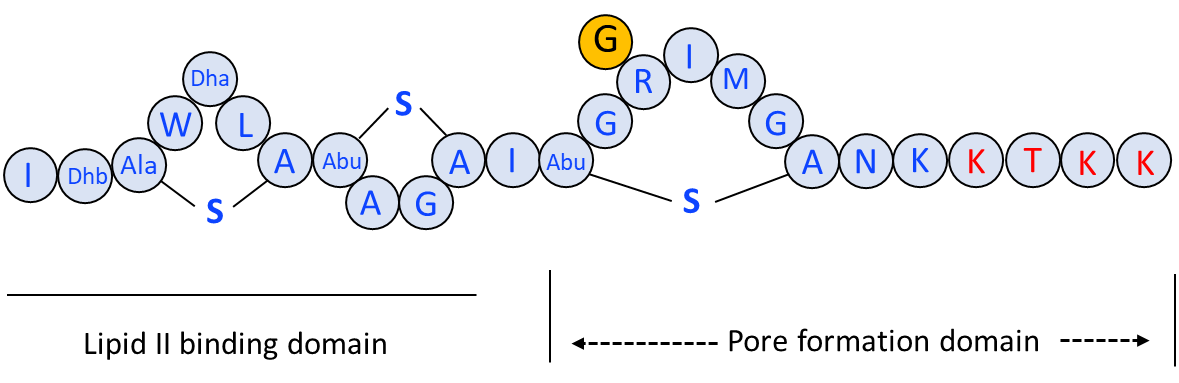


**A**

**B**

**Supplementary Figure 1**. Bioengineered cesin analogs with the plantiricin C pore forming domain. (A) Two lantibiotic hybrids, cesin-KTKK or cesin(R/G)-KTKK were engineered by fusing cesin or cesin R/G with plantaricin C(2-5), respectively. (B) Antimicrobial activity of the two bioengineered hybrids.
